# Progress and perspectives on DNA-enabled architectures for energy storage and conversion

**DOI:** 10.1016/j.isci.2026.117940

**Published:** 2026-11-05

**Authors:** Anjana Puthanpurayil Jayarajan, Samanth Kokkiligadda, Soong Ho Um

**Affiliations:** 1 School of Chemical Engineering, Sungkyunkwan University, Suwon 16419, Republic of Korea

**Keywords:** DNA-based materials, DNA nanotechnology, energy storage, energy conversion

## Abstract

The demand for efficient and sustainable energy technologies is driving the development of materials with precise structural control and tunable functionality. DNA, traditionally known as a biological molecule, has recently emerged as a versatile platform for designing advanced energy materials due to its programmability, self-assembly, and rich chemical functionality. This review examines the expanding role of DNA in energy storage and conversion systems, including batteries, supercapacitors, electrocatalysis, and soft energy devices. We highlight how DNA serves not only as a structural template but also as an active component that influences ion transport, charge transfer, and catalytic behavior. By analyzing recent progress through a structure-function perspective, this review outlines design principles that can guide the development of DNA-based energy materials. The integration of biomolecular programmability with electrochemical functionality positions DNA as a compelling platform for future energy systems.

## Graphical abstract

**Figure:**
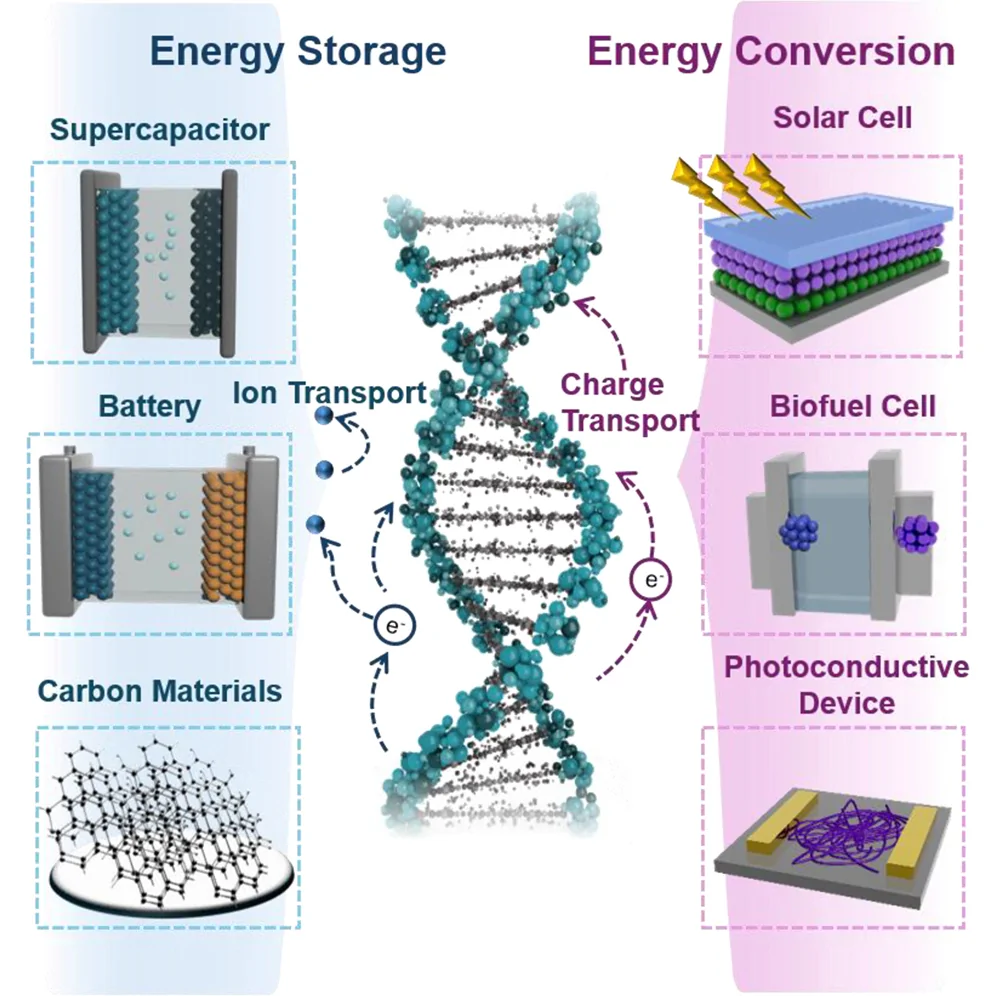

## Teaser

Nucleic acids; Bioengineering; Biomolecular engineering; Biotechnology; Nanotechnology; Energy storage; Energy materials

## Introduction

The escalating global demand for energy, fueled by population growth, urbanization, industrial expansion and the rapid electrification of transportation and infrastructure, has heightened the urgency for efficient, sustainable and high-performance energy storage and conversion technologies.^1^ Systems such as batteries, supercapacitors, and electrocatalytic devices play a central role in enabling renewable energy integration, grid stabilization, and next-generation portable and wearable electronics.^2^ While progress has been made using conventional inorganic and carbon-based materials, persisting challenges related to resource sustainability, structural limitations, and environmental impact continue to limit their long-term viability.^3^^,^^4^^,^^5^ Most state-of-the-art electrode and catalytic materials rely on fossil-derived precursors, synthetic polymers, or energy-intensive nanostructures such as graphene and carbon nanotubes.^6^^,^^7^^,^^8^^,^^9^ While these materials offer excellent conductivity and electrochemical performance, their production often involves high energy consumption, limited scalability, and environmental concerns.^10^^,^^11^ In addition, achieving precise control over their structure and functionality at the molecular level remains difficult, which indirectly limits the optimization of ion transport, active site distribution, and overall device efficiency. These challenges have raised interest in alternative material platforms which can combine sustainability and molecular-level design flexibility.

In this regard, biomolecules have provided a unique and unconventional class for functional materials.^12^ Among these, DNA has attracted considerable interest due to its intrinsic programmability, base-pairing interactions, and the possibility to assemble itself into well-defined architectures across multiple length scales.^13^^,^^14^^,^^15^ Initially developed as a nanotechnology and molecular engineering tool, DNA has been widely used as a templating framework for nanostructures, guiding the assembly of metallic and semiconductor components, and forming highly ordered frameworks with nanosized precision.^16^^,^^17^^,^^18^ These properties provide novel possibilities for the design of materials with controlled morphology and hierarchical porosity, as well as spatially defined functional sites, which are crucial for optimizing electrochemical processes.

Beyond its role as a structural scaffold, DNA has been increasingly investigated as a functional material.^19^^,^^20^^,^^21^^,^^22^ The ability of DNA to form functional species interacting with metal ions, its phosphate backbone, and its nitrogen-containing bases offer strong interactions with metal ions and other functional species and allow for the synthetic design of hybrid organic-inorganic systems.^23^^,^^24^^,^^25^^,^^26^ After thermal treatment, DNA can be converted into heteroatom-doped carbon materials containing nitrogen and phosphorus, which are well-known to improve electrical conductivity, catalytic activity, or surface reactivity. In parallel, DNA-based hydrogels and soft materials can be developed that have the ability to tune the transport of ions and show relative mechanical flexibility.^27^ These properties can be exploited to design biocompatible and flexible devices, and applications for aqueous batteries, flexible devices, and bio-integrated energy devices have been demonstrated.^28^^,^^29^^,^^30^ In this way, the recent progress in the use and conversion of DNA as a material has made possible the emergence of DNA-derived and DNA-enabled materials for a large range of energy applications. In the context of energy storage systems, DNA-derived carbons have shown promising performance as electrode materials in lithium-ion, sodium-ion, and zinc-based batteries, as well as in supercapacitors, where their hierarchical porous structure and intrinsic heteroatom doping have contributed to improved charge storage and ion diffusion.^31^^,^^32^^,^^33^ In the field of energy conversion, DNA has been exploited to template electrocatalysts with desired size and dispersion for enhancing their activity toward important reactions. In addition to these applications, the capability of DNA in mediating charge transfer and organizing functional molecules has also sparked an emerging interest in photoelectrochemical and bio-electrochemical systems.^34^^,^^35^^,^^36^^,^^37^

Nevertheless, the field of DNA-based materials is still relatively in an early stage of development. Most reported systems are based on proof-of-concept demonstrations, and more challenges remain before DNA-based materials can be translated into practical technologies. Especially, the stability of materials under electrochemical conditions, cost-effective and large-scale production of materials, and reproducibility of their properties are of key concern. Furthermore, because the underlying mechanisms of how DNA exerts influence on ion transport, charge transfer, and catalytic activities are not completely clear in many cases, the rational design for high-performance applications is limited. However, despite a growing body of research, critical challenges remain, including the need to integrate insights from various fields, from the molecular level to macroscale performance.

This review seeks to offer a unified and critical overview of DNA-based materials in energy storage and conversion. We emphasize the identification of how the structural and chemical properties of DNA can be harnessed to design functional materials, and how these properties can influence the function of key processes, from ion transport to charge storage to catalytic activity. Additionally, in order to tackle the emerging problems, some researchers have started investigating various methods that are proving to be quite promising. Chemical modifications to the DNA molecule and the application of protective coatings may increase the durability under electrochemical environments. A combination of DNA and inorganic structures, along with DNA and carbon, may provide increased mechanical robustness and electrical conductivity. Furthermore, the usage of genomic DNA from biowaste could serve as a cost-effective solution in comparison to artificially synthetic polymers. Improvements in the integration of devices at a smaller level would also facilitate compatibility with energy storage technology. These emerging strategies provide a clear pathway toward the large-scale implementation of DNA-enabled energy systems. This review provides a comprehensive overview of current advances in DNA-enabled energy systems while integrating developments in DNA nanotechnology, materials chemistry, and electrochemistry. We hope this review can guide the field by identifying current opportunities in the use of DNA as a functional materials platform, highlighting existing limitations in their application, and outlining potential directions for advancing these materials toward their fullest potential. In recent years a growing number of articles has been written about the design of energy storage and conversion systems in which they are active.^38^^,^^39^ Materials are based on the use and modification of DNA. These works have addressed many of the challenges identified above yet have often focused on specific applications or material types, or have touched upon issues of scalability, environmental impact, or sustainability, for example, in isolation rather than in integration. This makes it difficult to gain a holistic view of how the choice of functionalization or structure design can impact various aspects of a material from the atomic level up to the macroscopic level. As a result, current literature has often focused on individual applications, such as the development of biodegradable supercapacitor electrodes or enzymatic biomass-based fuel cells, rather than presenting a systemic view that integrates key insights from energy storage and conversion systems.

## DNA as a functional platform: A new classification

DNA is often considered a generally available material that can be used for various energy applications. However, the biological molecule is more than just a universal raw and building material. From a system-oriented point of view, it is more advantageous to think of the functions it can perform. The results from recent studies and analyses reveal that DNA is not simply treated as a passive object, but rather as an active component, which actively influences the structure, chemistry, and transport at various scales.^40^^,^^41^^,^^42^ In this commentary, we outline the three basic functional aspects of DNA as it can serve as a structural architect, as a transport medium, and as a transformation precursor. From this perspective, DNA-based energy materials can be designed from a holistic perspective and connect apparently many studies and applications.

### DNA as a structural architect

As soon as the double helix structure of DNA was identified, scientists recognized its ability to self-assemble. In fact, this property was recognized more than fifty years ago, but it was not until the introduction of DNA origami in 2006 that this ability could be used to make more complex two- and three-dimensional structures with near-molecular precision.^43^ DNA origami has since been used in nanotechnology and biophysics,^44^ but it has also drawn the attention of materials scientists who seek to understand and design new materials and material properties.^45^^,^^46^^,^^47^ As researchers have learned how to manipulate single-stranded and double-stranded DNA, they have begun to use it as a scaffold for the templated growth of inorganic materials.^48^^,^^49^^,^^50^ The resulting hybrid materials exhibit improved control over size distribution, morphology, and organization of metal, semiconductor, and insulating materials, in addition to biohybrid materials that combine both inorganic and organic components. The increased control is crucial for enhancing the active surface area of electrocatalysts through directed assembly of catalytic metal nanoparticles, which have been studied for use in fuel cells, electrochemical oxidation of pollutants, and photoelectrochemical water splitting.^51^ The improved assembly and structural control offered by DNA as a scaffold therefore enables precise modification of physical and electrochemical properties of resulting hybrid materials. DNA can also be used as a programmable blueprint to rationally design new materials with unique architectures, chemical compositions, and physical properties by selectively altering the DNA scaffold, such as by modulating sequence length or composition, or by employing various assembly approaches.

Recent work has explored the use of DNA origami-assembled Ag-coated Au nanostar (Au@Ag) core-shell structures for enhanced electrocatalytic water splitting. The study, led by G. Kaur et al., demonstrated electrocatalytic activity at the DNA Origami-assembled hybrid nanostructures for the oxygen evolution reaction (OER), outperforming both ss-DNA-functionalized and pure Au nanostars. Notably, the DNA Origami-assembled Au@Ag core-shell nanostructures showed enhanced activity for OER, reaching a current density of 10 mA cm^−2^ at an overpotential of 266 mV, surpassing the performance of state-of-the-art IrO_x_ electrocatalysts, which reached a current density of 5 mA cm^−2^ at an overpotential of 280 mV.^52^

### DNA as a transport medium

In addition to being a biological macromolecule, genetic material, DNA, can also be used to transport ions in a hydrated medium. DNA strongly interacts with cations via its polyanionic backbone and with water molecules. As a result, dynamic hydration shells are produced, capable of transporting ions.^53^^,^^54^ In soft materials, DNA can also assemble into a three-dimensional network-like structure (DNA hydrogel) that is able to hold a large amount of water, while still maintaining rigidity.^55^^,^^56^^,^^57^ In such materials, hydrated channels generated by the network-like structure of DNA function as ion conduction pathways, and the DNA-based hydrogels function as quasi-solid electrolytes in numerous applications,^58^ including biosensors, bio-batteries, fuel cells, and bio-supercapacitors.

Wu Yafeng et al. prepared a novel DNA hydrogel-integrated nanochannel membrane that exhibited enhanced ion flux and even ion rectification, which can be precisely controlled by external stimuli such as pH and ions.^59^ Its unique transport characteristics, where ions can move through the membrane in a specific direction, differ fundamentally from typical ion-conducting matrices. Such an active transport membrane is stable for long-term operation, with no significant electrolyte leakage or mechanical deterioration. The findings of this study have important implications for the ion transport within aqueous batteries, in particular for Zn-based systems, where the DNA network may act to suppress uncontrolled side reactions and enhance cycle life. Further work is needed to elucidate the exact transport mechanisms that are operative in these remarkable biohybrid membranes. Beyond ion transport, DNA can also mediate charge transport through its π-stacked nucleobases. Charge migration in DNA has been proposed to occur through two primary mechanisms: superexchange-mediated charge transfer, in which charges tunnel through the base stack over relatively short distances, and multistep hopping transport, where charges migrate sequentially between neighboring nucleobases over longer distances. The dominant mechanism depends on the energetic alignment of donor, bridge, and acceptor states, as well as the DNA sequence and base stacking interactions. The π-π overlap between adjacent nucleobases facilitates charge migration, enabling DNA to function as a molecular transport medium in electrochemical and bioelectronic systems.^60^

### DNA as a transformation precursor

DNA, a fundamental biomacromolecule, has been extensively explored as a versatile scaffold or matrix for the fabrication of diverse functional materials. It is increasingly recognized, however, that DNA can serve as a precursor to yield advanced energy materials, including fuels and energy storage and conversion devices, by providing a readily available nitrogen- and phosphorus-rich building block.^61^ Upon thermal treatment, the nucleobases and phosphate backbone of DNA can be converted into carbon and retain the embedded heteroatoms as covalent substituents within the resulting carbon framework, generating so-called heteroatom-doped (primarily N and P) carbon materials.^37^^,^^62^ This straightforward approach, which does not require any additional doping steps, offers a unique pathway to achieve high-performance carbon materials for energy applications in energy technologies. Importantly, the retained nitrogen- and phosphorus-containing functionalities can induce charge redistribution within the carbon matrix, generating catalytically active sites and facilitating charge-transfer processes. In addition, heteroatom doping can modulate the adsorption behavior of reaction intermediates, while the porous structure of DNA-derived carbons enhances electrolyte accessibility and active-site exposure.^63^ These combined effects improve electrode-electrolyte interactions, accelerate reaction kinetics, and enhance overall electrochemical performance. This strategy is particularly promising for the development of sustainable materials from biomasses and waste streams, since abundant and low-cost DNA resources are readily available. For instance, a simple hydrogelation-calcination protocol was employed to obtain porous carbon electrodes from biomass-derived DNA waste.^64^ The resulting material delivered a specific capacitance that outperformed commercial graphite electrodes. Such sustainable DNA-derived electrodes were integrated into energy storage devices to power electronic devices via a solar charging system. In summary, the findings highlight the vast potential of sustainable DNA-derived materials for energy applications. Importantly, they demonstrate that DNA is more than a simple scaffold or matrix; it can also serve as feedstock for the generation of functional materials with unprecedented properties and accessible scalability.

## DNA-enabled material design and functional integration

### DNA-templated material systems

One of the most direct ways DNA is employed in materials design is through templating. Owing to its predictable structure and programmability, DNA can guide the assembly of inorganic components such as metal nanoparticles and nanowires into well-defined arrangements.^65^ This enables precise control over parameters such as particle size, spacing, and distribution, which are critical for optimizing surface-dependent processes. Nucleic acid nanotechnology has established a foundation for designing DNA frameworks with high structural precision and addressability, making them ideal for organizing molecular components into complex assemblies.^66^^,^^67^^,^^68^ In this context, programmable DNA templates have been widely explored for fabricating inorganic and hybrid materials through techniques such as metallization, mineralization, lithography, and hierarchical self-assembly. Figure 1 presents a representative example of DNA-templated Cu_x_S nanoparticle synthesis and functionalization, illustrating the programmable role of DNA in directing nanomaterial assembly.^69^ These approaches highlight the versatility of DNA in directing material growth beyond conventional synthesis strategies. DNA templating has emerged as an effective strategy for the fabrication of metal nanoarchitectures through sequence-selective binding and reduction of metal ions, enabling the formation of nanoparticles, conductive nanowires, and organized assemblies.^70^ The intrinsic properties of DNA, including its ordered structure and chemical functionality, allow precise control over metal deposition, which is essential for developing functional nanomaterials for catalysis and electronic applications. Furthermore, advances in DNA nanotechnology have enabled the controlled synthesis of metal nanostructures with tunable morphologies using DNA-directed metallization processes.^71^ These methods provide enhanced control over growth pathways and structural features, allowing the fabrication of materials with tailored properties for applications ranging from catalysis to sensing and nanoelectronics. In many cases, DNA-templated systems exhibit enhanced electrochemical performance due to improved exposure of active sites and more uniform architectures.^39^ Unlike conventional synthesis approaches, DNA provides a level of spatial control that allows for more reproducible and tunable material design.

**Figure 1:**
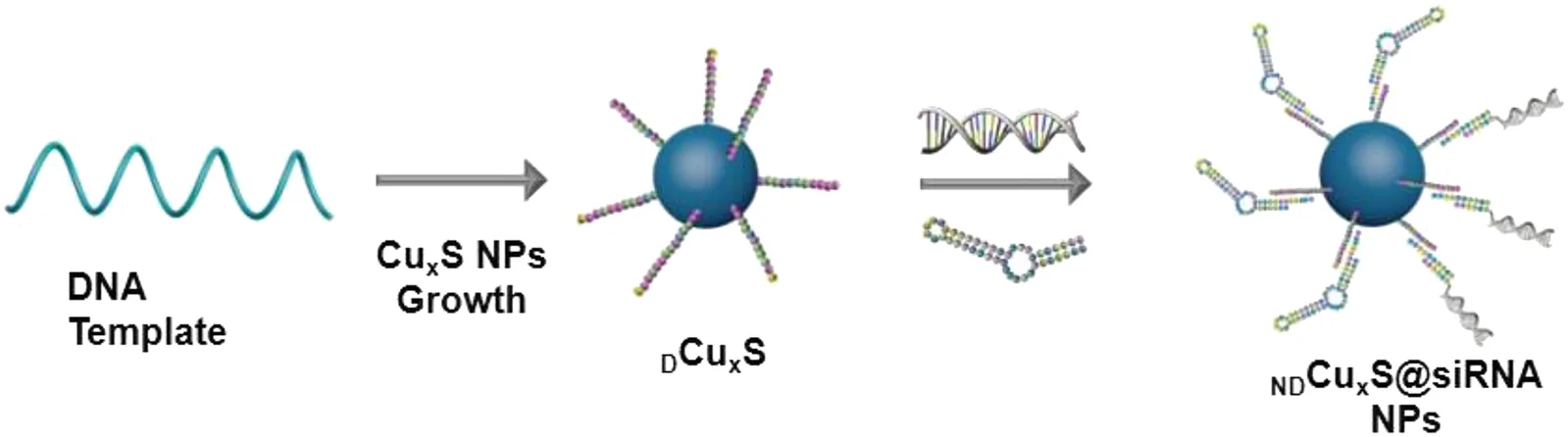
Presents a representative example of DNA-templated^69^ The DNA template directs Cu_x_S nanoparticle formation while enabling the incorporation of functional biomolecules through complementary base pairing.

### DNA-derived carbon materials

Beyond templating, DNA can also serve as a precursor for the synthesis of functional carbon-based materials. Through thermal treatment processes such as pyrolysis, DNA can be converted into carbon frameworks that inherently contain nitrogen- and phosphorus-rich functionalities derived from its molecular structure. This eliminates the need for additional doping steps, while enabling the formation of materials with high surface area, tunable porosity, and electrochemically active sites. In one study, DNA nanofibers were pyrolyzed to form carbon nanowires (CNWs) exhibiting excitation-dependent photoluminescence, demonstrating their potential for photo-induced energy and charge-transfer applications.^72^ The incorporation of Ag nanoparticles further enhanced fluorescence behavior, highlighting how DNA-derived carbon structures can be engineered for optoelectronic and energy-related functionalities. In another study, DNA was used to disperse double-walled carbon nanotubes (DWNTs), followed by thermal treatment to form freestanding, flexible electrodes.^73^ During this process, DNA was converted into phosphorus-enriched carbon, contributing additional redox-active sites, while the nanotubes provided high electrical conductivity. This synergistic combination enabled the development of bendable supercapacitor electrodes with improved electrochemical performance.

Correspondingly, one study reported porous carbon electrodes derived from biomass DNA waste via a hydrogelation-calcination process without additional activation.^64^ The resulting material exhibited a high specific capacitance of 563.34 F g^−1^ and demonstrated practical applicability by powering small electronic devices when integrated with a solar charging system. This highlights the potential of DNA as a sustainable precursor for scalable energy storage materials. Taken together, these studies demonstrate that DNA is not only a structural or functional component, but also a versatile feedstock for generating advanced carbon materials. The ability to directly convert a biological macromolecule into electrochemically active carbon frameworks provides a unique pathway toward sustainable and high-performance energy materials.

### DNA-inorganic hybrid systems

Another important design strategy involves the integration of DNA with inorganic materials to form hybrid systems. In these architectures, DNA interacts with metal ions,^74^ metal oxidesm^75^ or mineral phases^76^ through electrostatic interactions, coordination, or molecular recogniti, leading to the formation of structurally organized composites. Such systems benefit from the complementary properties of both components, where the inorganic phase contributes conductivity or catalytic activity, while DNA provides structural organization and chemical functionality. A representative study demonstrated that DNA can mediate the crystallization of inorganic magnesium pyrophosphate (Mg_2_PPi) through rolling circle amplification and long-strand templating approaches, resulting in densely packed DNA-inorganic hybrid composites.^77^ The presence of flexible DNA chains influenced nucleation and growth behavior, enabling controlled incorporation of biomolecular components such as enzymes while maintaining structural integrity (Figure 2). This demonstrates how DNA can actively regulate inorganic crystal formation rather than simply serving as a passive scaffold.

**Figure 2:**
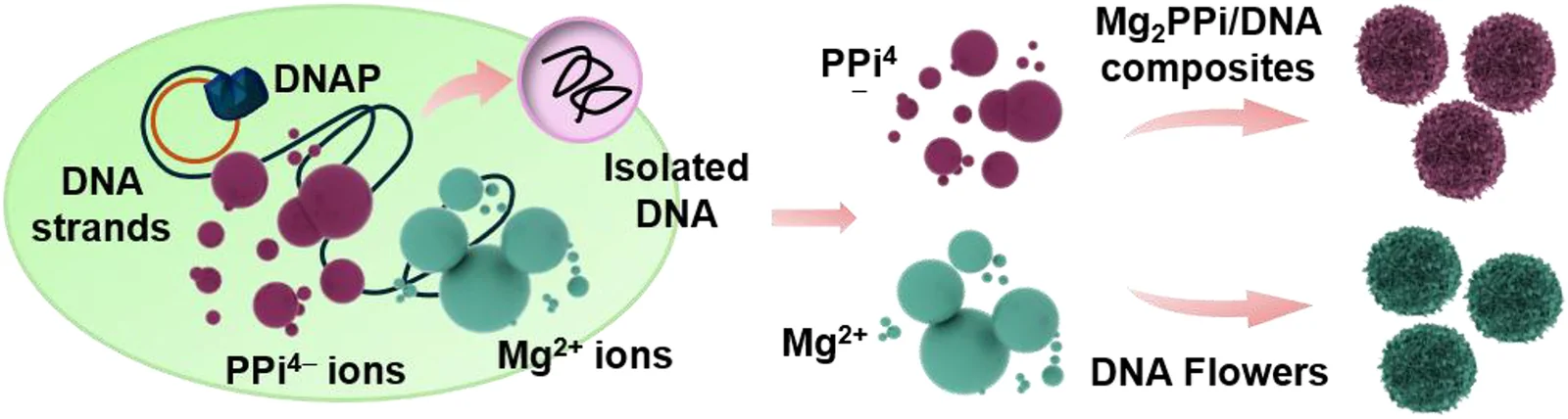
DNA-inorganic hybrid systems^77^ Schematic illustration of the interaction of DNA with pyrophosphate and Mg^2+^ ions, leading to the formation of Mg_2_PPi/DNA composite structures and DNA flowers.

In another study, researchers explored structural DNA-inorganic hybrid materials formed through the integration of DNA with nanoparticles such as gold, iron oxide, and quantum dots.^78^ These systems exhibited synergistic behavior arising from the combination of programmable DNA architectures and functional inorganic components, enabling improved performance across applications including sensing, catalysis, and bio-integrated technologies. Such hybrid systems highlight the versatility of DNA in bridging soft and hard matter, enabling the design of materials where structure, composition, and functionality are tightly coupled. This ability to direct interactions at the interface makes DNA-inorganic hybrids a powerful platform for developing next-generation materials for energy and related applications. By virtue of this combination of DNA with other inorganic elements, another important form that has evolved is DNA-based hydrogels, which extend the functionality and structure of DNA even more. The hydrogels provide a pathway to enhance scalability and practical applicability while maintaining the inherent programmability of DNA.

### DNA hydrogels as functional materials

DNA hydrogels represent a distinct class of soft materials where structural organization and transport properties are inherently coupled. Formed through the crosslinking of DNA strands into three-dimensional networks, these hydrogels are capable of retaining large amounts of water while maintaining mechanical integrity. This combination of structural stability and hydration makes them particularly relevant for electrochemical applications, where both ion transport and mechanical robustness are critical. Recent developments have increasingly shifted toward the use of naturally derived genomic DNA from biowaste sources, offering a more sustainable and scalable alternative to synthetic DNA systems. These hydrogels exhibit inherent sequence diversity and tunable network architectures, enabling control over ionic conductivity, flexibility, and responsiveness to environmental stimuli (Figure 3). As a result, they are being actively explored as components in batteries, supercapacitors, and other energy storage systems.

**Figure 3:**
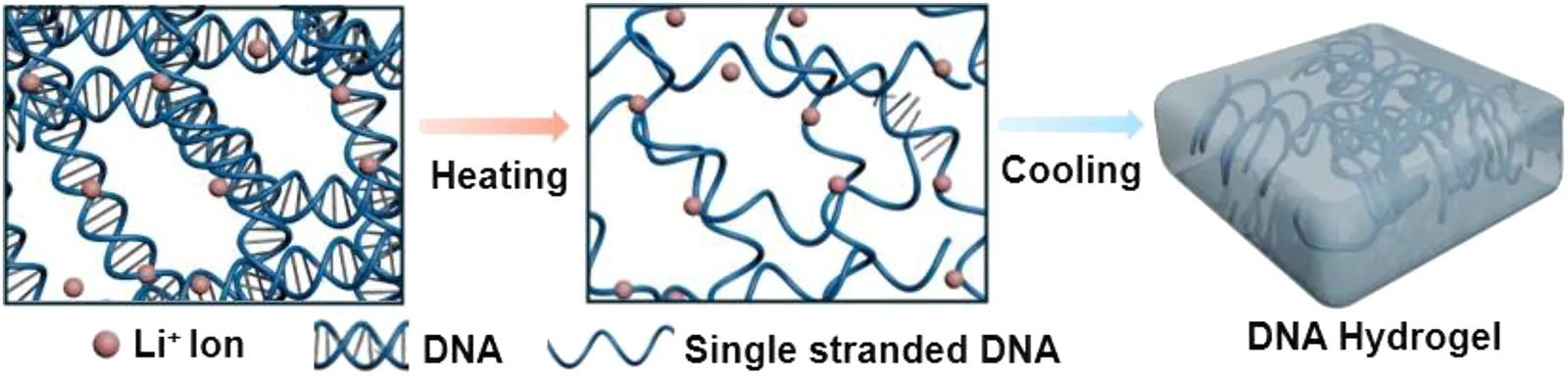
DNA-based hydrogels^79^ Schematic illustration of DNA hydrogel formation through heating-induced strand dissociation followed by cooling and reorganization of the DNA network in the presence of Li^+^ ions.

A genomic DNA-based hybrid gel electrolyte with significantly improved mechanical strength and ion transport properties, achieving high ionic conductivity and electrochemical stability, was reported.^79^ When applied in supercapacitor devices, the system demonstrated high specific capacitance, excellent cycling stability over extended cycles, and competitive energy and power densities. This highlights how DNA hydrogels can move beyond structural roles to function as active electrochemical components. A subsequent study reported that a three-dimensional DNA hydrogel was used as an electron transport scaffold by incorporating redox-active mediators within its network. The resulting system enabled long-range electron transfer beyond the limitations of conventional two-dimensional electrodes, demonstrating how DNA hydrogels can facilitate both ionic and electronic transport within a single platform.^80^ Together, these studies illustrate that DNA hydrogels are not merely passive matrices but multifunctional materials capable of integrating mechanical support, ion conduction, and even electron transport. This combination of properties positions them as promising candidates for next-generation energy storage and bio-integrated electronic systems.

### Structure to function: Integration pathways

The effectiveness of DNA-based materials ultimately depends on how structural design translates into functional performance. Rather than treating DNA as a passive material, recent approaches increasingly view it as a programmable building block capable of directing material synthesis with high precision.^81^ This perspective has led to the emergence of a gene-like construction paradigm, where DNA sequence, structure, and assembly pathways are deliberately designed to control material properties across different length scales. At the molecular level, sequence-defined interactions enable precise tuning of chemical functionality.^82^ At the nanoscale, DNA directs the assembly of hybrid complexes with polymers, small molecules, and inorganic components. At the macroscale, DNA hydrogels provide structurally robust and functionally adaptable frameworks. Such an approach allows for rational material design, where properties such as conductivity, catalytic activity, and ion transport can be systematically engineered. For instance, templated systems enhance surface-controlled reactions, DNA-derived carbons introduce active sites, and hydrogel-based systems enable efficient ion and electron transport. By integrating these design principles, DNA-based materials can be developed in a more targeted and predictable manner. This shift from empirical design to programmable construction represents an important step toward the scalable and application-driven use of DNA in energy systems.

## DNA-enabled architectures for energy storage

In recent years, the integration of DNA nanotechnology into energy storage systems has emerged as a highly promising and rapidly evolving research area (Table 1). This is primarily due to the unique physicochemical properties of DNA, including its precise structural programmability, molecular recognition capability, tunable surface chemistry, and inherent biocompatibility. Unlike traditional materials used in electrochemical devices, DNA offers an exceptional level of control over nanoscale organization, which enables the rational design of advanced architectures for improved energy storage performance. Importantly, DNA does not merely function as a passive structural component, but it actively participates in defining the morphology, connectivity, and interfacial properties of the resulting materials. What makes DNA stand out in energy storage is how it shapes electrode materials at microscopic levels. In this context, DNA-templated synthesis has proven to be an effective strategy for producing well-defined nanostructures. During fabrication, using DNA as a guide leads to precise nanoscale patterns forming reliably. For example, a DNA-assisted hydrothermal approach has been successfully employed to fabricate Bi_2_MoO_6_ nanoplate structures with superior uniformity and regularity compared to their non-templated counterparts.^83^ The presence of DNA during synthesis regulates nucleation and growth processes, resulting in more controlled architecture. This structural refinement significantly enhances electrochemical behavior, as evidenced by a high specific capacitance of 698 F g^−1^ and notable cycling stability, with 86% capacitance retention after 3000 cycles. Such improvements clearly demonstrate the effectiveness of DNA in tailoring material properties for supercapacitor applications. DNA-assisted synthesis has also been employed to fabricate nickel-cobalt sulfide (NiCo_2_S_4_) nanosheets featuring well-defined porous networks.^84^ Depending on how much DNA is added, the shape and pore layout shift noticeably. The optimized nanosheets delivered a high specific capacity of 644C g^−1^ at the current density of 1 A g^−1^, superior rate capability of 74.3% retention at 15 A g^−1^ and remarkable cycling stability of 90.5% after 1500 cycles, highlighting the critical role of DNA in engineering electrode architectures with enhanced electrochemical performance (Figure 4A). It turns out DNA does not just carry genetic code; rather, it steers how tiny building blocks arrange themselves. Beyond morphology control, DNA helps shape hybrid electrode systems. Instead, it guides how materials come together, especially when linked to carbon nanotubes coated with DNA strands. These hybrids, called CNTs@DNA, protrude due to their ability to combine the excellent electrical conductivity of CNTs with the functional versatility of DNA. DNA wraps around CNTs using π-π stacking interactions. At the same time, its hydrophilic sugar-phosphate backbone improves the dispersion in liquid environments. Because of this dual functionality, the conductive networks formed are stable and well-dispersed. One example involves multi-walled carbon nanotubes coated with DNA, used as scaffolds for growing NiCo_2_O_4_ nanoparticles, leading to the formation of NiCo_2_O_4_-CNT@DNA structures.^85^ Because of its structure, DNA improves how evenly CNTs spread out while also acting as starting points for consistent particle development (Figure 4B). Owing to these features, the resulting electrode shows a high specific capacitance of 760 F g^−1^ and excellent cycling stability, with over 96% capacitance retention after 5000 cycles. When built into asymmetric supercapacitor setups, such materials achieve notable levels of both energy and power delivery and can power light-emitting diodes, demonstrating their practical applicability.

**Table 1:** Comparison of representative DNA-enabled energy storage systems

References	DNA-enabled material/system	Electrolyte	Loading	Current density/Rate	Cycling	Practical performance metrics
Yesuraj et al.^83^	DNA-templated Bi_2_MoO_6_ nanoplates	1M NaOH	3 mg	2 A g^−1^	3000 cycles	high specific capacitance with excellent cycling stability
Liu et al.^84^	DNA-assisted NiCo_2_S_4_ nanosheets	6M KOH	1 mg cm^−2^	2 A g^−1^	1500 cycles	high specific capacity and excellent rate capability
Xue et al.^85^	DNA-directed NiCo_2_O_4_/CNT electrode	3M KOH	NR	5 A g^−1^	5000 cycles	high capacitance and outstanding cycling stability
Li et al.^86^	DNA-guided Li_2_S nanostructure cathode	1 M LiTFSI/LiNO_3_in DME/DOL (1:1, v/v)	10 mg cm^−2^ sulfur	0.1C	100 cycles	8.1 mAh cm^−2^ areal capacity, enhanced sulfur utilization
Ouyang et al.^87^	programmable DNA interphase layer	ether and carbonate electrolyte	DNA layer: 0.01 mg cm^−2^	1 mA cm^−^^^2^^	425 cycles	Coulombic efficiency of 99.1%, uniform Li deposition, high reversibility
Chilamkurthi et al.^88^	DNA-assisted defect-rich MnO_2_ cathode	2M ZnSO_4_ +0.2M MnSO_4_ electrolyte	1.14 mg cm^−2^	10 A g^−1^	10,000 cycles	high-rate capability and long cycling life
Kim et al.^89^	DNA-wrapped CNT conductive binder	1M LiPF_6_	7 mg cm^−2^	1C/3C	700 cycles	improved electrode integrity and cycling stability
Kim et al.^90^	DNA-metallized anode	1M LiPF_6_ dissolved in EC/DEC (1:1, v/v)	–	1.4C	150 cycles	improved lithium-storage performance

**Figure 4:**
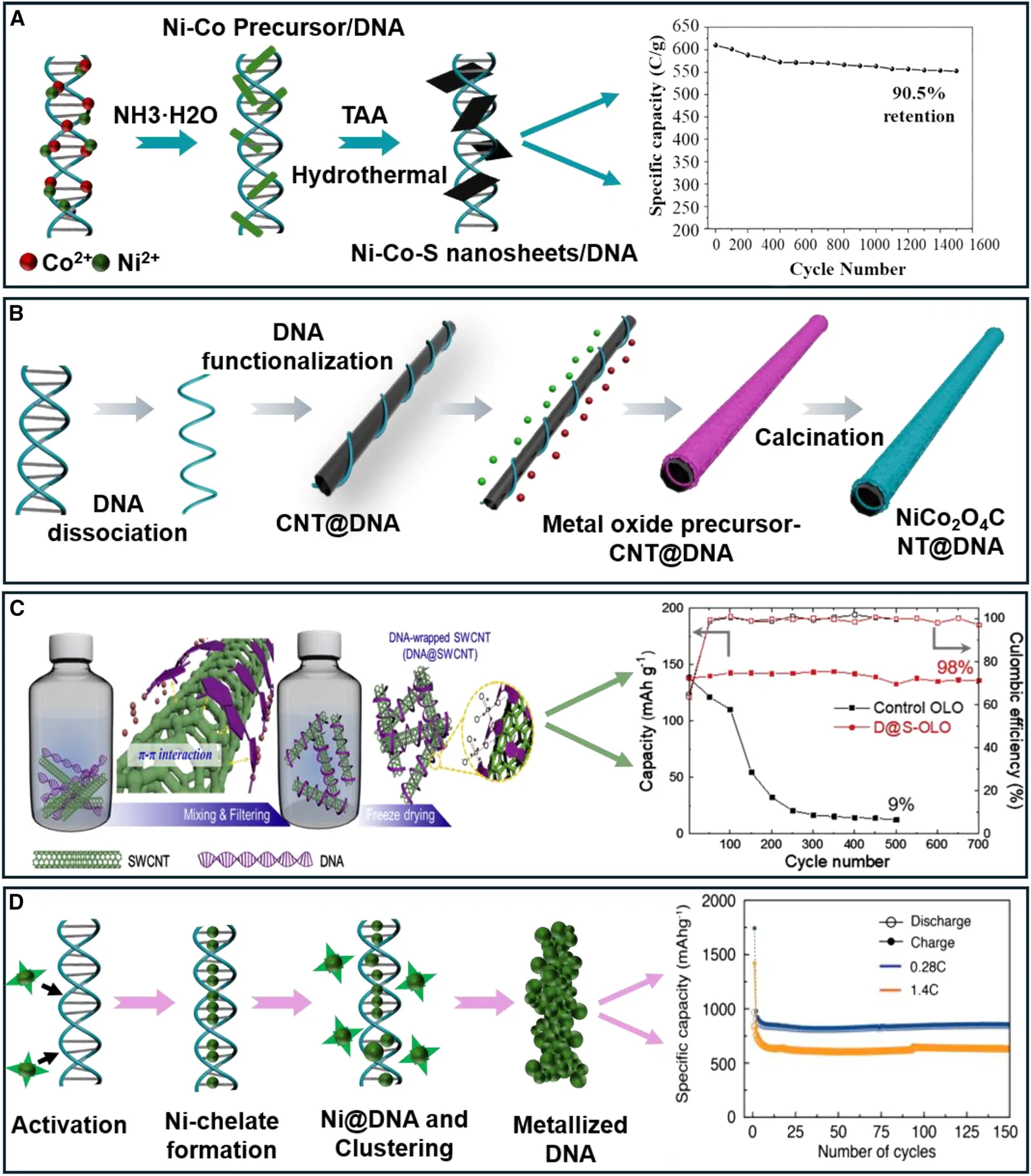
DNA-enabled materials for advanced energy storage applications (A) DNA-assisted synthesis of NiCo_2_S_4_ nanosheet electrodes. Reprinted with permission from Hanmeng et al.^84^ Copyright 2018 Elsevier. (B) DNA-wrapped multi-walled carbon nanotube (MWCNT) hybrid system.^85^ (C) DNA-wrapped single-walled carbon nanotube (SWCNT) conductive binder network. Reprinted with permission from Kim et al.^91^ Copyright 2020 Elsevier. (D) DNA-templated nickel oxide (NiO) one-dimensional nanostructures. Reprinted with permission from Kim et al.^86^ Copyright 2014 Elsevier.

Among recent strategies, carbon nanotubes modified with DNA bridge manganese dioxide spheres into a connected framework known as CNTs@DNA-MnO_2_.^33^ In this setup, DNA does more than just stabilize; rather, it spreads out nanotubes evenly while guiding MnO_2_ into organized clusters. Because these components align closely, electrons move faster across the network. The DNA-assisted assembly and electrochemical mechanisms were experimentally validated using complementary characterization techniques, including SEM and TEM for morphological analysis, XPS for confirming DNA functionalization and chemical interactions, and CV, galvanostatic charge-discharge (GCD), and electrochemical impedance spectroscopy (EIS) for evaluating charge storage behavior, kinetics, and cycling performance. This interconnected structure enhances charge transfer kinetics and ensures efficient utilization of active materials, resulting in improved energy density of 11.6 W h kg^−1^ attained at a power density of 185.5 W kg^−1^ and overall device performance in asymmetric supercapacitors. Starting with unconventional approaches to energy storage, researchers have turned to DNA-based platforms for more than just charge retention. Going further from traditional electrochemical storage methods, DNA materials are also being considered for multifunctional and self-powered systems. One such innovation is the use of MoS_2_ nanoparticles and carbon quantum dots with DNA materials to create multifunctional nanocomposites.^87^ Such materials have the ability to harvest energy through triboelectric, sense motion, and store energy. In addition, the generated systems produce large output voltage values (of up to 1.6 kV) and high-power densities. Moreover, the systems can also act as capacitors. The performance of these nanocomposites can be controlled by external stimuli, such as light and temperature. Such multifunctional behavior highlights the potential of DNA-based materials for next-generation wearable and implantable electronic devices.

DNA-based methods have made notable progress in improving battery systems, especially where controlling ion transport and interfacial reactions are critical. Lithium-sulfur-based batteries often underperform due to their poor sulfur utilization and the formation of insulating Li_2_S layers. To tackle such problems, researchers employed DNA, which uses its negatively charged backbone to guide the nucleation and Li_2_S growth.^88^ Here, during the polysulfide conversion in the battery, the DNA-decorated carbon nanosheets promote the Li_2_S nucleation along the DNA chains, as the negatively charged phosphate groups in the DNA sugar-phosphate backbone electrostatically attract the Li^+^ ions. Because of this guidance, the deposition forms a three-dimensional Li_2_S architecture rather than a dense insulating layer, boosting the pathways for ions and electrons without sacrificing reactive areas. The proposed mechanism was experimentally validated using complementary characterization methods. SEM observations at different discharge states directly revealed the transition from a compact Li_2_S layer on the control electrode to three-dimensional Li_2_S agglomerates on the DNA-modified cathode, while EDS elemental mapping confirmed the coexistence of DNA and deposited Li_2_S. Polysulfide adsorption tests and DFT calculations demonstrated the strong interaction between DNA functional groups and Li_2_S_8_, whereas CV and EIS confirmed improved Li_2_S conversion kinetics and charge transport. Consequently, batteries with high sulfur loading achieve significantly improved areal capacities of 8.1 mAh cm^−2^, reaching values more than twice those of conventional lithium-ion batteries. In addition, DNA has been utilized as a programmable interphase layer in anode-free lithium metal batteries.^89^ The molecular structure of DNA, where the abundant nitrogen-containing nucleobase pairs regulate homogeneous Li^+^ flux by forming transient Li-N interactions. These interactions promote a homogeneous Li^+^ flux across the electrode surface, leading to uniform and compact lithium deposition while suppressing localized Li accumulation and dendritic growth. As a result, the DNA interphase enhances the reversibility and kinetics of Li plating/stripping, stabilizes the electrode-electrolyte interface, and leads to improved reversibility and cycling stability, even under high current densities. The proposed mechanism was experimentally validated using complementary techniques. Spectroscopic analyses, microscopic observations (SEM, TEM, STEM), surface characterization (TOF-SIMS and XPS), and electrochemical measurements collectively confirmed the DNA-mediated regulation of Li^+^ flux, stable SEI formation, and improved Li plating/stripping behavior. The resulting systems exhibit high coulombic efficiency of 99.1% over 400 cycles and long cycle life, demonstrating the potential of DNA as a novel interfacial engineering material. Other than lithium-ion battery chemistries, DNA-assisted technologies have also shown great promise for use in aqueous zinc-ion batteries.^90^ Using the DNA-assisted hydrothermal technique, δ-MnO_2_ nanoflake electrodes can be synthesized to be rich in defects by virtue of the addition of DNA, which introduces phosphate and nitrogen functional groups in the material, forming complexes with Mn ions to increase interlayer space, Mn^3+^ content, and defects. The presence of such characteristics in electrode materials enhances the kinetics of Zn^2+^ diffusion and storage. As a result, the DNA-functionalized manganese dioxide cathode offers a specific capacity of 293.3 mAh g^−1^ at 0.2 A g^−1^ and still maintains an impressive capacity of 121.6 mAh g^−1^ at 10 A g^−1^.

One innovative application of how DNA supports batteries lies in acting as a conductive binder. By replacing conventional polymer binders, DNA-wrapped single-walled carbon nanotubes (DNA@SWCNT) have been developed as nanofibrous binders.^91^ As these DNA-based binders link components tightly, they enhance electrical conductivity, improve adhesion to current collectors, and provide better ion transport pathways. As a result, the OLO (overlithiated layered oxide) cathode exhibited faster charging capability reporting an 83% relative capacity ratio after 15min of charging. Moreover, the system showcased high-capacity retention of 98% even after a prolonged 700 cycles as well as improved thermal stability (Figure 4C). Furthermore, DNA metallization represents a powerful approach for constructing conductive nanostructures. Starting with DNA’s natural negatively charged backbone, continuous metallic structures along DNA template are formed due to the selective deposition of metal ions, resulting in the fabrication of one-dimensional nickel oxide nanostructures.^86^ This fabricated unique 1D DNA structures contributed a high reversible capacity of 850 mAh g^−1^ and robust cycling performance for 150 cycles (Figure 4D). Such nanostructures effectively accommodate volume changes during charge-discharge cycles, thereby improving battery durability. Ending on a practical note, DNA-guided materials play roles in the applications in energy-related sensing technologies. Palladium nanoparticles synthesized on DNA templates exhibit unique hydrogen absorption properties, making them suitable for hydrogen sensing applications.^92^ These Pd-DNA hybrid nanostructures show high sensitivity and reversible response due to volume changes associated with hydrogen absorption, illustrating how DNA-based systems can bridge energy storage and sensing functionalities.

Overall, the above examples clearly demonstrate that DNA serves as a highly versatile and multifunctional material platform in energy storage applications. It enables precise control over nanostructure formation, enhances material dispersion and connectivity, improves interfacial properties, and facilitates the development of multifunctional systems. Despite challenges related to scalability, stability, and cost, the unique advantages offered by DNA make it a promising candidate for future sustainable and high-performance energy storage technologies.

## DNA-enabled architectures for energy conversion

DNA-based materials are increasingly being explored in energy conversion systems, where they contribute not only as structural elements but also as active functional components. Unlike conventional materials, DNA offers a unique combination of programmability, molecular recognition, and charge transport capability, allowing it to participate in processes such as light harvesting, electron transfer, and catalytic reactions. As a result, DNA-enabled architecture is emerging as promising platforms for converting solar, chemical, and mechanical energy into electrical energy.

One of the most significant applications of DNA in energy conversion lies in photovoltaic devices. Recent studies have shown that DNA can function as an effective charge transport medium, particularly for hole transport. For example, in a perovskite-based system, a core-shell structure was developed using DNA modified with cetyltrimethylammonium chloride (DNA-CTMA).^93^ This design not only improved hole extraction and transport but also enhanced the overall device performance, achieving a power conversion efficiency of 20.63%. In addition, the hydrophobic nature of the DNA-CTMA layer helped stabilize the device, retaining over 90% of its initial efficiency after prolonged exposure to ambient conditions (Figure 5A). Interestingly, a simplified version of the device without a conventional hole transport layer significantly reduced fabrication cost while maintaining high efficiency. In another approach, DNA nanostructures were used as electron-blocking layers in polymer solar cells.^95^ The introduction of a two-dimensional DNA lattice improved charge separation and reduced recombination losses, resulting in an increase in power conversion efficiency ranging from 10% up to 160%. In addition, incorporating DNA into the electron transport layer of perovskite solar cells helped reduce trap states and improve band alignment, leading to a notable enhancement in efficiency from 13.25% to 17.59% (Figure 5B).^94^ These examples clearly demonstrate how DNA can improve both the performance and stability of photovoltaic systems.

**Figure 5:**
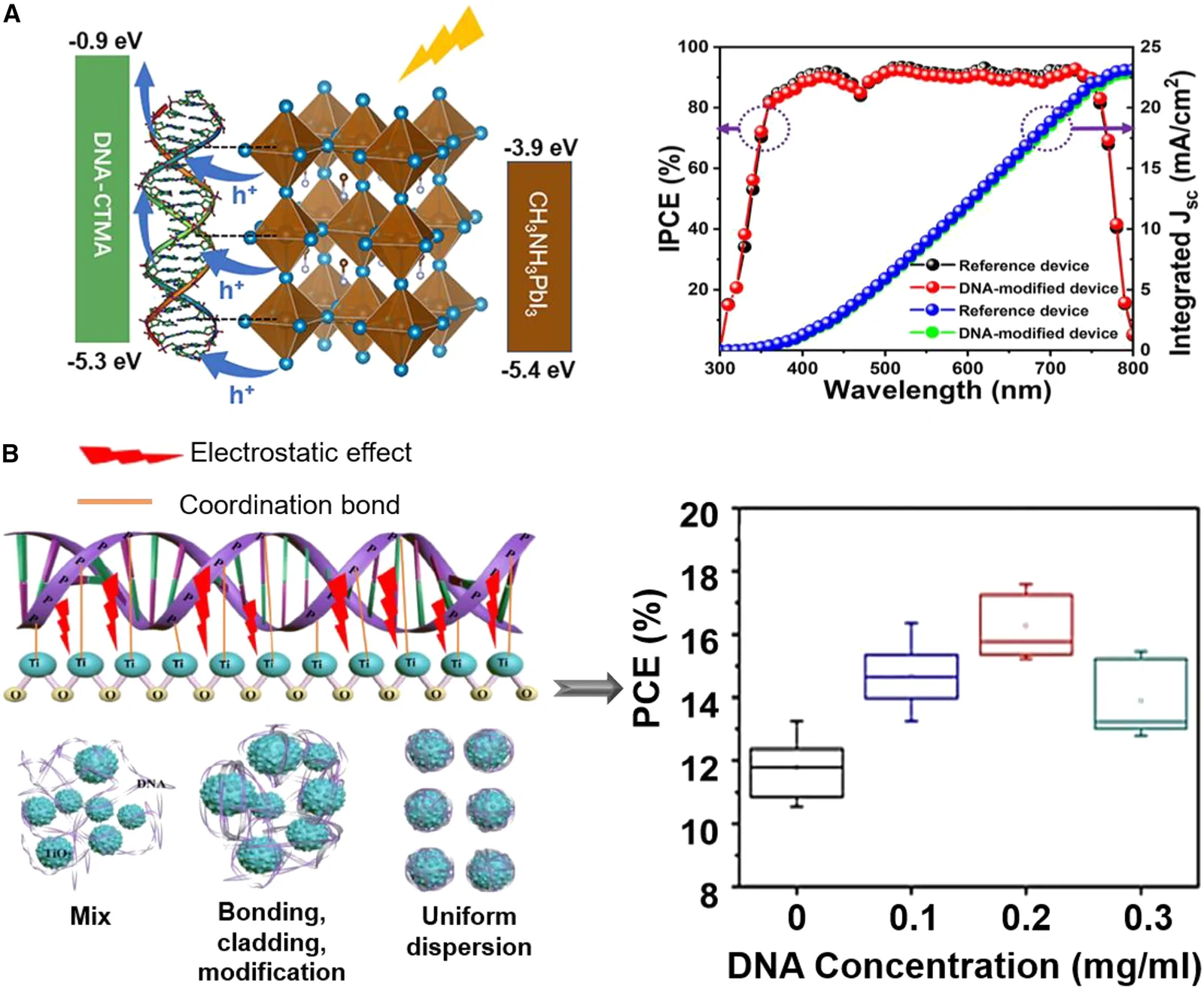
DNA-enabled strategies for enhancing photovoltaic performance (A) Core-shell perovskite architecture employing DNA-CTMA as a hole transport layer, enabling efficient hole extraction and transport, achieving a power conversion efficiency (PCE) of 20.63% and retaining more than 90% of initial efficiency under ambient conditions, while also enabling low-cost hole-free device configurations. Reprinted with permission from Yuchen Hou et al.^93^ Copyright 2020 Elsevier. (B) DNA-doped mesoporous TiO_2_ electron transport layer in perovskite solar cells, reducing trap state density and carrier recombination, resulting in improved band alignment and enhanced PCE. Reprinted with permission from Xian et al.^94^ Copyright 2020 Elsevier.

Beyond photovoltaics, DNA-based materials also show strong potential in photoconductive and optoelectronic applications. Due to its ability to organize nanomaterials into well-connected networks, DNA can facilitate efficient charge and energy transfer. For instance, a DNA-molybdenum disulfide (MoS_2_) nanocomposite film exhibited exceptionally high photoconductivity.^96^ The system achieved a photocurrent of 5.5 mA at 1 V and an on/off ratio of 1.3 × 10^6^, which is significantly higher than typical biomaterial-based systems. The photoresponsivity reached 2.6 × 10^5^ A W^−1^, indicating its strong capability for light-to-electricity conversion. Furthermore, DNA-templated supramolecular assemblies enabled efficient energy transfer through a multistep hopping mechanism.^97^ These systems showed a quantum yield of 0.40, highlighting the ability of DNA to guide excitation energy transport over molecular distances. Such properties make DNA-based architecture highly attractive for advanced optoelectronic devices. DNA also plays a crucial role in biofuel cells, where it helps enhance enzyme stability, electron transfer, and overall device efficiency. In one example, DNA-wrapped single-walled carbon nanotubes were used to immobilize enzymes in a biofuel cell operating in serum conditions.^98^ This configuration protected the enzyme activity and enabled stable power generation of around 190 μW/cm^2^ for up to one week (Figure 6A). Another interesting development involves DNA-based self-powered biosensors that integrate enzymatic biofuel cells with DNA amplification strategies.^100^ These systems demonstrated extremely high sensitivity, with detection limits as low as 20 a.m. The improved performance is mainly due to the ability of DNA to facilitate efficient charge transfer and amplify electrochemical signals. Additionally, DNA-templated metal nanoclusters, particularly gold nanoclusters, have been explored as cocatalysts for oxygen reduction reactions, which are essential in biofuel cell operation.^101^ This further highlights the versatility of DNA in catalytic energy conversion processes. Another important aspect of DNA-enabled energy conversion lies in its ability to direct the formation of catalytic and hybrid nanostructures. By guiding the formation of nanostructures with controlled size, shape, and distribution, DNA enables the development of highly efficient catalytic platforms. For example, DNA-templated silver and gold nanoclusters exhibit tunable optical and catalytic properties, including enhanced fluorescence and improved catalytic activity.^101^ In another approach, DNA-assembled nanoparticle lattices were converted into silica-based replicas that retained their structural organization even under extreme conditions such as high temperature and pressure (Figure 6B).^99^ This significantly improves the durability of DNA-based systems, making them suitable for practical energy applications. More broadly, DNA-directed assembly has been used to construct nanowires, hybrid nanostructures, and functional composites that combine the of both biological and inorganic materials.^102^ These hybrid systems enable better control over electron transport pathways and catalytic activity, which are critical for efficient energy conversion.

**Figure 6:**
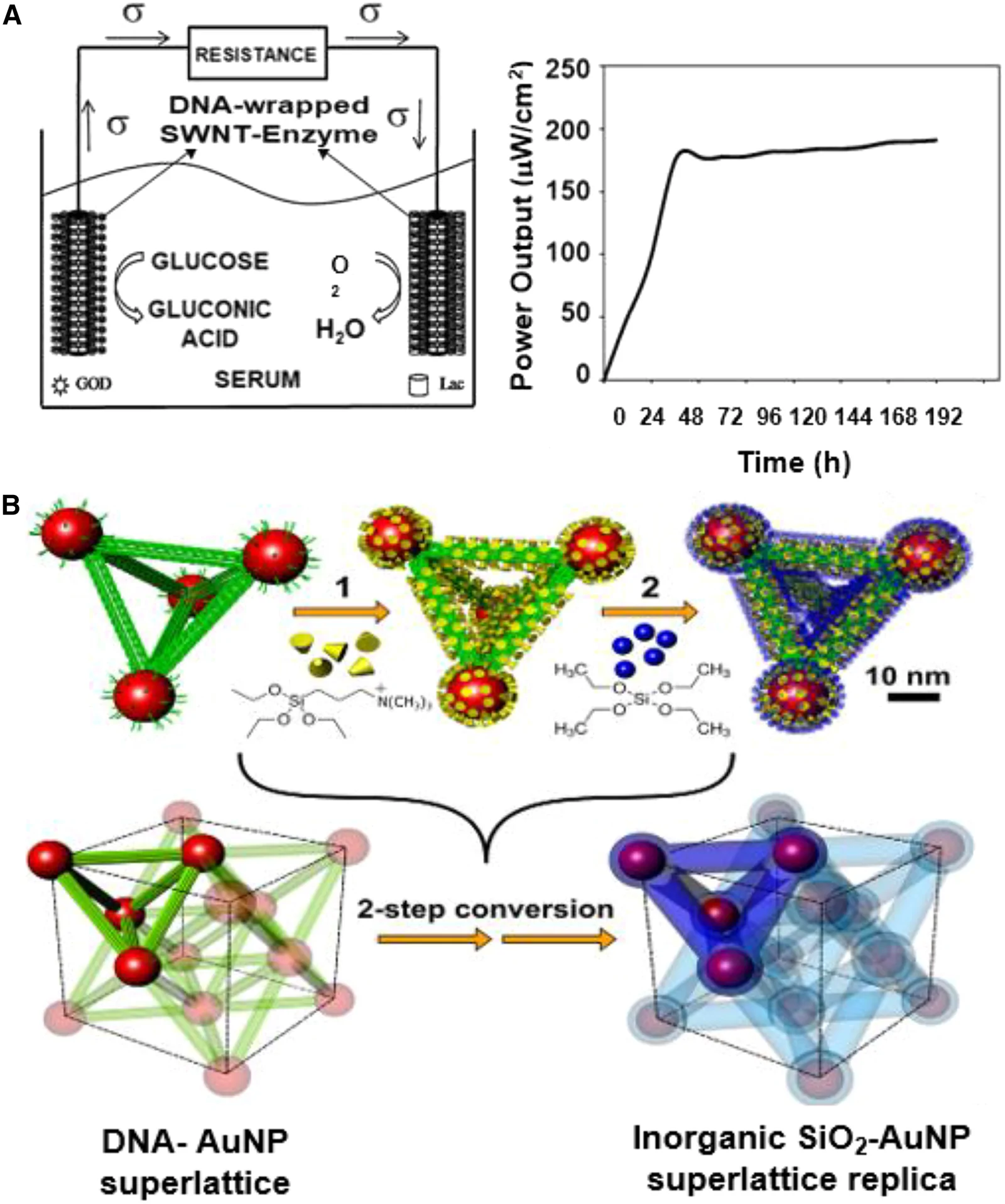
DNA-enabled architecture for bio-electrochemical systems and robust nanomaterial fabrication (A) DNA-wrapped single-walled carbon nanotube (SWNT) based bioelectrode in an enzyme-based biofuel cell, enhancing enzyme stability and electron transfer under serum conditions, delivering a sustained power output. Reprinted with permission from Lee et al.^98^ Copyright 2011 Elsevier. (B) Silica-replicated 3D DNA-templated nanoparticle lattices, preserving the structural topology of DNA-assembled frameworks while significantly improving mechanical and environmental stability. Reprinted with permission from Majewski et al.^99^ Copyright 2021 American Association for the Advancement of Science.

DNA-enabled architecture contributes to energy conversion in multiple interconnected ways. They can act as charge transport pathways, structural templates, stabilizing matrices, and even active participants in energy transfer processes. The ability to precisely design and control these functions at the molecular level gives DNA a clear advantage over many conventional materials. With demonstrated improvements in efficiency, stability, and functionality across photovoltaic devices, biofuel cells, and optoelectronic systems, DNA-based materials are gradually establishing themselves as a promising platform for next-generation energy technologies. While challenges such as scalability and long-term durability remain, ongoing research continues to address these limitations through hybrid material design and advanced fabrication strategies.

## Bottlenecks in DNA-enabled architectures for energy storage and conversion

Although DNA-enabled architectures have demonstrated significant promise across both energy storage and conversion systems, their practical implementation remains constrained by several fundamental limitations. A major bottleneck arises from the difficulty in translating molecular-level precision into scalable material systems. DNA-based architectures rely on highly controlled assembly processes that are efficient at small scales but challenging to reproduce uniformly in bulk production. As system size and structural complexity increase, the precision of molecular interactions becomes harder to maintain, leading to reduced assembly yield and increased variability. Additionally, larger-scale fabrication often requires more resources and processing steps, contributing to higher production costs.^19^ As a result, maintaining structural fidelity and consistent functional performance at scale remains a significant challenge, limiting their practical applicability in real-world energy devices.^58^ Furthermore, the practical implementation of DNA enabled materials depends on the type of DNA employed. Naturally derived DNA is more sustainable and readily available but may exhibit variations in molecular weight, sequence composition, and purity, which can affect reproducibility. In contrast, sequence-specific synthetic DNA, such as DNA origami, offers superior programmability but requires complex synthesis and purification, resulting in significantly higher production costs. Therefore, balancing programmability, reproducibility, and economic feasibility remains a key challenge for the large-scale deployment of DNA-enabled energy materials.^103^

Another critical issue is the limited operational stability of DNA under electrochemical conditions. Exposure to fluctuating temperatures, reactive species, and prolonged cycling can disrupt the structural integrity of DNA, leading to degradation of performance over time. While hybridization with other materials can enhance robustness, it often introduces additional complexity and compromises the intrinsic advantages of DNA. From a functional standpoint, charge transport remains a key limitation. Despite its ability to organize nanoscale components, DNA itself is not an inherently efficient conductor. This necessitates the incorporation of conductive additives or nanomaterials, which can lead to interfacial losses, aggregation issues, and reduced control over architecture. Economic feasibility also presents a significant challenge. The cost associated with DNA synthesis, processing, and purification, combined with the need for precise sequence design, makes large-scale deployment difficult. Even when alternative biological sources are explored, issues related to standardization and reproducibility persist.

Finally, integration into complete energy systems remains underdeveloped. Bridging the gap between soft, biomolecular architectures and conventional device components such as electrodes and current collectors which requires further innovation to ensure compatibility, durability, and performance reliability. In addition, most reported DNA enabled energy systems have been evaluated under laboratory conditions rather than practical operating environments. Many studies employ low active material loadings, excess electrolyte, and half-cell configurations, whereas commercially relevant devices require high areal loading, lean electrolyte operation, limited alkali metal inventory, pouch cell validation, and long-term cycling stability. Systematic evaluation under these practical conditions will be essential to assess the true commercial potential of DNA-enabled energy materials.

## Future perspectives for DNA-enabled energy systems

The future of DNA-enabled architecture lies in overcoming these bottlenecks through innovative, interdisciplinary approaches that combine materials science, biotechnology, and device engineering. One of the most promising directions is the development of hybrid and multifunctional systems, where DNA acts as a programmable scaffold integrated with conductive and catalytic materials. Such systems can leverage the structural precision of DNA while enhancing electrical and electrochemical performance, enabling more efficient energy storage and conversion. Advancements in sustainable and scalable DNA sourcing will also play a crucial role. Utilizing naturally derived DNA or developing biotechnological production methods could significantly reduce costs and enable large-scale fabrication, making DNA-based systems more commercially viable. As summarized in Table 2, biomass DNA extracted from biological waste provides a practical route for linking cost reduction with environmental protection, because waste-derived DNA can be transformed into functional materials while reducing dependence on expensive purified DNA and lowering the environmental burden of conventional feedstocks. Compared with conventional DNA production methods such as PCR, rolling circle amplification (RCA), cell culture, and sequence-specific synthesis (e.g., DNA origami)^106^^,^^107^, biomass-derived DNA offers greater potential for cost-effective and scalable production, although highly programmable DNA architectures remain comparatively expensive for large-scale applications. For example, biomass-DNA-based plastics have been demonstrated using DNA from algae, fish, plants, and food waste, and the reported conversion cost at lab scale was about USD 1 per gram, with potential large reductions at industrial scale. In contrast, conventional DNA production methods such as PCR,^104^ RCA, and cell culture^105^ are often limited by higher costs, lower bulk yields, or additional processing requirements, making them less advantageous than biomass-derived DNA for large-scale application. In addition, although DNA stability remains a practical concern, approaches such as chemical fixation, encapsulation, and hybridization with inorganic materials have shown promise in enhancing the long-term durability of DNA-enabled energy materials.

**Table 2:** Comparative overview of conventional DNA production methods, including PCR, rolling circle amplification (RCA), and cell culture

Method	Typical output	Expected cost	Advantages	Disadvantages	References
PCR	in one reported workflow, PCR-based assembly produced a 2.4 kb gene construct	about USD 700–800 for all routes in that study, depending on oligo design	fast, flexible, and useful for generating defined DNA fragments	less efficient for bulk DNA production and often requires error correction for longer constructs	Xiong et al.^104^
RCA	one cited workflow produced 10 μg DNA in 16 h	about USD 4 per reaction, or about USD 0.3 per μg	very low cost per mass, high fidelity, and isothermal operation	usually needs circular templates and may require product cleanup for some uses	Marano et al.^105^
Cell culture	the cited bacterial/plasmid workflow produced about 80 μg DNA	about USD 175–650 total, or about USD 2–8 per μg	familiar, scalable, and practical for standard propagation	slower, more labor-intensive, and more expensive per μg than RCA	Marano et al.^105^
DNA origami/sequence-specific synthesis	typically prepared at nanogram-to-microgram scale per design batch, depending on scaffold and staple usage.	substantially higher than bulk DNA routes because of oligonucleotide synthesis, purification, and assembly costs.	exceptional programmability, addressability, and structural precision.	high production cost, complex purification, and limited scalability for bulk deployment.	Praetorius et al.^106^; Yang et al.^107^
Bio-derived/extracted DNA	scalable source material for materials use, but output depends strongly on extraction yield and purity.	often lower feedstock cost than fully synthetic DNA, but purification and batch variability can add cost.	more sustainable and readily available at large scale.	batch-to-batch variation in molecular weight, purity, and composition can affect reproducibility	Liao et al.^103^

Another key opportunity is the evolution of DNA from a passive structural component to an active functional material. By introducing redox-active groups, ion-conducting pathways, or dynamic binding sites, DNA architectures can directly participate in energy storage and conversion processes, enhancing overall device efficiency. The integration of DNA-based materials with smart and adaptive technologies represents a transformative frontier. DNA’s programmability opens possibilities for responsive systems capable of self-regulation, performance optimization, and real-time sensing. Such features could lead to intelligent energy devices that adapt to operational conditions and extend device lifespan. Sustainability will remain a defining advantage of DNA-enabled systems. The use of biodegradable and renewable materials aligns with global efforts toward environmentally responsible energy technologies. Future research may focus on designing fully recyclable or degradable energy devices, further enhancing their ecological impact. The key advantages of DNA-enabled energy systems, including tunability, sustainability, biocompatibility, and the potential for improved stability through material engineering, are summarized in Figure 7.

**Figure 7:**
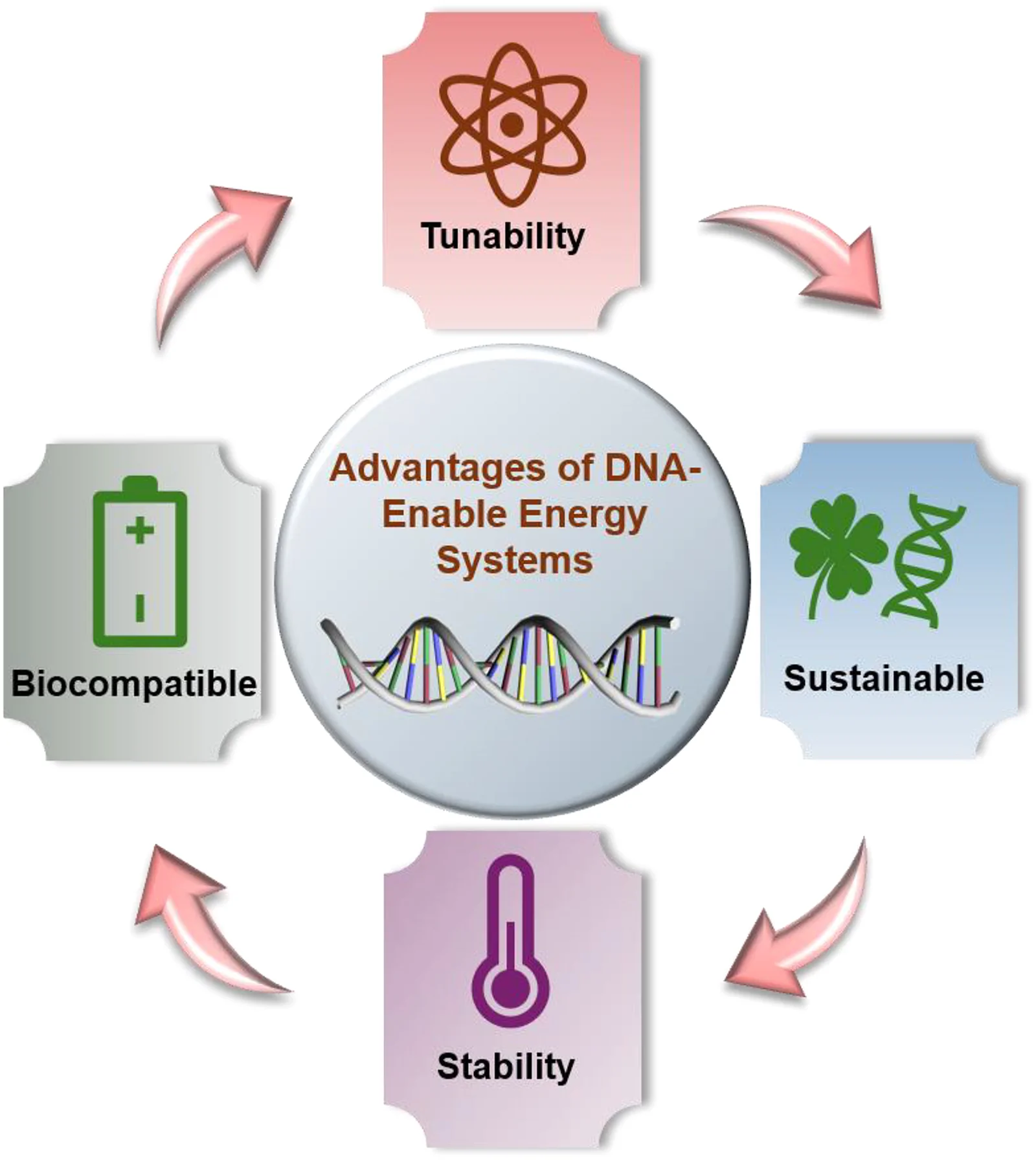
Advantages of DNA-enabled energy systems Schematic illustration highlights the key features of DNA-enabled energy systems, including tunability, sustainability, stability, and biocompatibility.

## Conclusion

DNA-enabled architecture represents a rapidly evolving paradigm in the field of energy storage and conversion. Their unique ability to precisely organize materials at the molecular level offers unprecedented control over structure-function relationships, enabling innovations that are difficult to achieve with conventional materials. Despite the progress made, challenges related to scalability, stability, conductivity, and cost continue to limit their widespread adoption. Addressing these issues will require a concerted effort to develop robust, scalable, and economically viable systems without compromising the intrinsic advantages of DNA. Looking ahead, the convergence of DNA nanotechnology with advanced materials, sustainable sourcing strategies, and intelligent system design is expected to unlock new possibilities for next-generation energy technologies. As these developments mature, DNA-enabled architectures have the potential to move beyond proof-of-concept studies and become integral components of practical, high-performance, and sustainable energy systems.

## Acknowledgments

This work was supported by the 10.13039/501100003725National Research Foundation of Korea and the 10.13039/501100014188Ministry of Science and ICT (RS-2025-09382983, RS-2026-25470732 and RS-2026-25587162).

## Author contributions

A.P.J.: writing - original draft, investigation, validation, visualization, and formal analysis. S.K.: supervision, project administration, resources, writing – review and editing, and validation. S.H.U.: supervision, funding acquisition, project administration, resources, writing – review and editing, and validation. All authors reviewed and approved the final manuscript.

## Declaration of interests

The authors declare no conflict of interest.
